# Quantity and Quality Matter: Different Neuroanatomical Substrates of Apathy in Alzheimer’s Disease and Behavioural Variant Frontotemporal Dementia †

**DOI:** 10.3390/brainsci15050447

**Published:** 2025-04-25

**Authors:** Luciano Inácio Mariano, Thiago de Oliveira Maciel, Henrique Cerqueira Guimarães, Leandro Boson Gambogi, Antônio Lúcio Teixeira Júnior, Paulo Caramelli, Leonardo Cruz de Souza

**Affiliations:** 1 Programa de Pós-Graduação em Neurociências, Universidade Federal de Minas Gerais (UFMG), Belo Horizonte 31270-901, MG, Brazil; lucianoi.mariano@gmail.com (L.I.M.); leandroboson@gmail.com (L.B.G.); caramelli@ufmg.br (P.C.); 2 Behavioural and Cognitive Neurology Uni, Universidade Federal de Minas Gerais (UFMG), Belo Horizonte 30130-110, MG, Brazil; hcerqueirag@gmail.com; 3 Quantum Research Center, Technology Innovation Institute, Abu Dhabi P.O. Box 9639, United Arab Emirates; 4 Neuropsychiatry Program, Department of Psychiatry and Behavioral Sciences, McGovern Medical School, The University of Texas Health Science Center at Houston (UTHealth), Houston, TX 77054, USA; alterxr@gmail.com; 5 Santa Casa BH Ensino e Pesquisa, Belo Horizonte 30150-221, MG, Brazil; 6 Departamento de Clínica Médica, Faculdade de Medicina, Universidade Federal de Minas Gerais (UFMG), Belo Horizonte 30130-100, MG, Brazil

**Keywords:** apathy, frontotemporal dementia, Alzheimer’s disease, MRI, differential diagnosis, neuropsychology

## Abstract

Background: Apathy is almost ubiquitous across neurodegenerative diseases and can be a general model for understanding neuropsychiatric symptoms in dementia. Methods: We assessed apathy via Starkstein’s Apathy Scale—caregiver version (SAS-C) in patients with Alzheimer’s disease (AD) and behavioural variant frontotemporal dementia (bvFTD). Neuropsychological and structural neuroimaging data were also collected. Images were processed using the FreeSurfer program, and cortical thickness data were acquired for 68 brain regions. Results: Patients with bvFTD had statistically higher levels of apathy than those with AD. The multivariate linear regression model found that the left entorhinal cortex (lEC) was the only region statistically associated with apathy in the AD group (F(1,31) = 5.17; *p* = 0.030; R2 = 0.527), whereas, for bvFTD, the right lateral orbitofrontal cortex achieved significant association with apathy (F(1,30) = 5.69; *p* = 0.009; R2 = 0.804). Conclusions: These results demonstrate that apathy is associated with multiple brain regions, reinforcing its multidimensionality and specific profiles.

## 1. Introduction

Apathy is characterised by an impairment in implementing goal-directed behaviours, which, in fact, involves disturbances in multiple systems, such as cognitive, emotional, and motor processing [1]. In light of this, the updated criteria for apathy highlight its multidimensional spectrum [2,3], but the evidence to properly define which dimensions make up the syndrome is still debated [4].

In the field of dementia, the prevalence rates for apathy are 54% and 59% in the mild and moderate stages of dementia, respectively [5], and apathy affects functionality, quality of life, and prognosis [6].

Patients with Alzheimer’s disease (AD) and patients with behavioural variant frontotemporal dementia (bvFTD) are commonly diagnosed as apathetic, but they might not share the same type or dimension of apathy [7]. Wei et al. [8], for example, showed that apathy varies according to the disease stage, with early bvFTD exhibiting a predominant type of emotional apathy than that seen in AD. In the late stages, dysexecutive/cognitive apathy predominates in AD compared to bvFTD.

In order to better understand the phenomenology of apathy, neuroimaging studies have investigated brain regions and hubs associated with apathy. Using the Lille Apathy Rating Scale (LARS), Fernández-Matarrubia et al. [9] found that bvFTD had higher levels of “blunting of emotional responses” and “extinction of self-awareness” than AD. In bvFTD, the LARS scores inversely correlated with the left insular and left middle frontal gyri, while no correlation was found in the AD group. When using the clinical diagnosis criteria available at the time, as proposed by Robert et al. [10], they observed an association between apathy and the anterior cingulate.

Kumfor et al. [11] also investigated structural magnetic resonance imaging (MRI) and voxel-based morphometry (VBM) in a group of patients with AD and bvFTD. The authors used different questionnaires to gather data from three putative apathetic dimensions: affective, behavioural, and cognitive. Affective apathy was associated with ventral prefrontal cortex (PFC) regions, including the frontal medial cortex, anterior cingulate cortex (ACC), and left frontal pole, in patients with AD and bvFTD. Cognitive apathy was associated with more dorsal PFC regions, including the paracingulate gyrus, bilateral frontal operculum cortex, and superior frontal gyrus. This highlights how specific apathetic dimensions are associated with different regions of the brain, yet such specificity is not fully understood.

In a previous study, we observed that apathy measured with the caregiver version of Starkstein’s Apathy Scale (SAS-C) correlates with the orbitofrontal cortex (OFC) and the ACC in patients with bvFTD [12]. However, we were not able to compare bvFTD with AD to check if there are any different profiles in SAS-C brain networks. This is highly relevant given that SAS-C is an easy and frequently used instrument; therefore, understanding its brain correlates might be useful to indicate the specific mechanisms involved in apathy. Moreover, additional cognitive data and their association with apathy are presented here as part of an effort to better capture the phenomenon.

## 2. Materials and Methods

Fifty-nine participants were recruited for this cross-sectional study: 20 patients with bvFTD, 19 patients with AD, and 20 community-dwelling cognitively healthy older adults as controls. Groups were matched for age and educational level, and patients were also matched for symptom duration.

Patients were recruited from the Cognitive and Behavioural Neurology Unit at Belo Horizonte (University Hospital from the Federal University of Minas Gerais, Belo Horizonte, Brazil). Patients fulfilled consensus diagnostic criteria for probable bvFTD [13] or probable AD [14], and all were examined at the mild to moderate stages of dementia. Patients were clinically followed for at least 12 months after diagnosis to improve diagnostic accuracy, yet data were collected solely transversely, after the first formal diagnosis. All patients showed clinical progression consistent with their diagnosis, according to the consensus formed by the assisting health professionals.

Controls were included under the following criteria: (i) age older or equal to 55 years; (ii) mini-mental state evaluation (MMSE) results compatible with the mean for their age and educational level, according to the Brazilian norms [15]. Controls were excluded in the presence of neurological disorders, such as brain injury, strokes, seizures, or any psychiatric diagnosis, such as depression, anxiety, or other conditions. General health conditions that could preclude study participation also led to exclusion, e.g., kidney failure or severe heart disease.

All participants underwent a comprehensive neuropsychological evaluation and an MRI. Details are provided below.

### 2.1. Neuropsychological Evaluation

All participants underwent a standard neuropsychological protocol composed of the following tests: (i) MMSE [16] for global cognitive screening; (ii) the Frontal Assessment Battery (FAB) [17] for executive domains; (iii) the Digit Span Test (forward and backward) [18] for verbal short-term memory and verbal working memory; (iv) the Figure Memory Test (FMT) [19] to evaluate memory and its components: learning, storage, late recall, and recognition; (v) verbal fluency (FAS and animals) [20,21]; (vi) the Hayling Test [22] for verbal inhibitory control; and, (vii) the short version of the Social Cognition and Emotional Assessment (Mini-SEA) [23], composed of the Faux-Past Test (hereafter solely named Faux-Pas) to assess theory of mind (ToM); and the Facial Emotion Recognition Test (FERT) to investigate emotion recognition.

As aforementioned, apathy was assessed using the SAS-C [24]. The SAS-C comprises a 14-question third-person questionnaire about daily activities, engagement, and interests. A family member or caregiver answers it, avoiding any potential effects of anosognosia [25]. Controls answered a self-report version (SAS-S) composed of 14 self-centred questions.

### 2.2. Neuroimaging

Participants underwent whole-brain MRI on a 3 T Philips scan. T1-weighted images were acquired: multishot 256 TFE factor (TR/TE 5.4/2.4 ms, 256 × 256 matrix, FOV 256 × 256 × 180, flip angle 8°), 1 mm slice thickness, coronal orientation, and 1 × 1 × 1 mm^3^ voxel size. The MRI data were preprocessed following a standard protocol, as described elsewhere [26].

Cortical thickness (CTh) and subcortical volume estimations were obtained with Freesurfer v7 (http://surfer.nmr.mgh.harvard.edu, accessed on 23 May 2022). The preprocessing pipeline used the fully automated “recon-all” command. It included normalisation, removal of non-brain tissues, Talairach transforms, segmentation, and tessellation of grey matter (GM) and white matter (WM) boundaries [27]. The cortical surface of each hemisphere was parcellated according to the atlas proposed by Desikan et al. [28], with 34 cortical regions per hemisphere (“aparc” segmentation). Cortical thickness was estimated as described elsewhere [29,30,31]. Subcortical volumes were obtained via a whole-brain automatic “aseg” segmentation procedure [27].

Although we did collect subcortical data, due to the high number of variables in the regression model, we chose not to include it in our analysis. Incorporating this information would exceed the capacity of the statistical model, compromising its reliability. Hence, given the nature of the diseases we are investigating, we focused on cortical data. Future studies with larger samples may allow for a more comprehensive investigation of subcortical structures.

### 2.3. Statistical Analyses

All statistical analyses were performed using the software SPSS 22 (SPSS Inc., Chicago, IL, USA). Descriptive statistics were used to characterise groups. The Shapiro–Wilk test and visual inspection of histograms determined whether variables were under a normal distribution and the parametric or non-parametric test definition for further analysis. The non-parametric Kruskal–Wallis test was used to compare variables across the three groups of participants. Dunn’s post hoc test was employed when suitable to perform paired comparisons, applying Bonferroni correction and establishing *p*-value significance at 0.000735.

Given the non-normal distribution of data, the effect size was calculated by using the eta squared. The accuracy of using the SAS-C to differentiate the clinical groups from each other was checked by receiver operator curve (ROC) analysis, using a cutoff of 14 points [24].

Correlation analyses (Spearman’s test) were conducted between apathy scores and the CTh for each region. We explored correlations within two groups, composed of controls with each of the clinical groups (bvFTD or AD); hence, correlations were investigated for AD + controls (*n* = 39) and bvFTD + controls (*n* = 40). This approach has been adopted elsewhere [32,33].

Following the identification of significant correlations, multiple logistic regression was conducted using the assembled groups as previously described. Due to some cognitive tests showing statistically significant differences between groups, these were included in the model to prevent spurious associations. Specifically, the following variables were controlled for: MMSE total score, FAB total score, phonemic verbal fluency, Faux-Pas total score, and FMT immediate recall and late recall. As part of this exploratory analysis, we employed the stepwise model [34] and analysed regions separately per hemisphere.

## 3. Results

### 3.1. Sociodemograpgic and Cognitive Outcomes

The sociodemographic results and cognitive data are described in Table 1. There were no differences regarding age or education among all groups, and the symptom duration was similar between the clinical groups.

General cognition measurements were similar between the clinical groups, with MMSE and FAB separating controls from the others (AD = bvFTD < controls). Overall, the bvFTD group had a more dysexecutive profile than the AD group. Mnemonic performance was worse in AD. The Faux-Pas and the FERT had similar results for social cognition measures, with patients with bvFTD underperforming the control and AD groups.

Apathy was significantly higher within the clinical groups, but the highest levels were found in bvFTD (bvFTD > AD > controls). The accuracy of SAS-C scores for differential diagnoses between the AD and bvFTD groups was tested, and the ROC curve analysis showed an area under the curve (AUC) of 0.805 (*p* = 0.001; confidence interval: 0.66 to 0.95) (Supplementary Materials Table S1; Supplementary Figure S1).

### 3.2. Brain Regions Associated with Apathy

The correlation results for all regions can be found in the Supplementary Materials (Tables S2–S6).

#### 3.2.1. The Alzheimer’s Disease (AD) Group

Spearman correlation values showed significant *rho* coefficients from −0.323 to −0.619 between SAS-C scores and several brain regions. After Bonferroni’s correction for statistical significance, six regions from the right hemisphere and thirteen from the left hemisphere were included in the multiple regression model.

The final result indicated that only the MMSE score was statistically associated with SAS-C (F(1,37) = 6.51; *p* < 0.001; R2 = 0.432). Among the brain regions, only one area was statistically associated with the SAS-C score, namely the left entorhinal cortex (lEC) (F(1,31) = 5.17; *p* = 0.030; R2 = 0.527).

The full data can be seen in Supplementary Table S7.

#### 3.2.2. The Behavioural Variant Frontotemporal Dementia (bvFTD) Group

Spearman correlation values showed significant *rho* coefficients from −0.317 to −0.702 between SAS-C scores and several brain regions. After Bonferroni’s correction for statistical significance, 13 regions from the right hemisphere and 11 from the left hemisphere were included in the model. The multiple regression showed the association between some cognitive components and the SAS-C: the MMSE (F(1,36) = 8.31; *p* < 0.001; R2 = 0.499); the phonemic verbal fluency test (F(1,34) = 6.98; *p* < 0.001; R2 = 0.637); and the Faux-Pas test (F(1,33) = 6.27; *p* = 0.005; R2 = 0.708).

Regarding brain regions, only the right lateral orbitofrontal cortex (F(1,30) = 5.69; *p* = 0.009; R2 = 0.804) was associated with apathy (Supplementary Table S8).

## 4. Discussion

In the present study, we found that apathy, as measured by the SAS-C, was statistically higher in bvFTD compared to AD. The neuroanatomical substrates were also different. While the SAS-C correlated specifically with the lEC in AD, bvFTD correlates specifically with the lOFC. This is the first investigation to assess cortical thickness in patients with bvFTD and AD and to map brain differences using this instrument.

One must acknowledge that the variability of the areas involved in the apathy phenomenon could result from the instrument chosen to evaluate it, reflecting a plurality in the very conception of apathy. In the present study, we tested whether the SAS-C, a “simple” questionnaire, could differentiate bvFTD from AD from clinical and neuroanatomical points of view. The goal was to enhance the available knowledge on apathy by using this classical tool, allowing a deeper understanding of the phenomenological differences between apathetic patients with AD and those with bvFTD.

At first, we confirmed evidence showing that the degree of apathy is much higher in bvFTD than in AD, as shown by Fernández-Matarrubia et al. [9] using a different instrument. The accuracy found in our study reinforces the quantitative approach for separating bvFTD from AD, with the latter presenting lower levels of apathy. Hence, a higher degree of apathy may indicate a higher chance of bvFTD, although we naturally recognise that from a clinical point of view, other factors must be integrated to conclude a differential diagnosis.

From the neuroimaging analysis, our model proved valid enough to discriminate specific cortical brain regions associated with apathy in each condition.

The lEC was associated with apathy in AD. As part of the parahippocampal formation, the EC is involved in the complex process of memory consolidation. Evidence from studies working with major depression highlights the role of the EC in regulating hippocampus neurogenesis [35]. Although several brain regions are associated with memory consolidation, the particular interrelation between the EC and the basolateral amygdala highlights the role of emotional processing in mnemonic formation [36]. Remarkably, the lEC seems to be involved in experiencing connection, disconnection, or loneliness in older adults [37]. Hence, disturbances in the finetuning of emotional processing and memory storage may be associated with difficulties in engagement and motivation toward different activities. We must acknowledge that this study is limited with regard to lacking proper data to control for depressive symptoms in our sample. However, the typical signs of depressed mood (such as guilt, sadness, crying, and pessimism) were never part of the clinical presentation of the participants enrolled during the 12 months of follow-up after diagnosis.

For bvFTD, apathy was associated with the lOFC only. The role of the OFC in apathy and social restriction has been described over time in different clinical presentations, including in patients with dementia [38,39,40]. The OFC values potential reinforcers to sustain or dismiss a goal-directed behaviour [41]. While the medial OFC (mOFC) is more associated with the valuation of outcomes, the lOFC is responsible for dynamically modifying associations between stimuli and their effects, regardless of reward [42]. OFC hypoactivation can be implicated in more passive/non-reactive behaviour with the mediation of the ACC [43]. Although our results did not achieve statistical significance for the ACC, it is useful to acknowledge that the right ACC (rACC) also participates in risk calculation and avoidance [44]. Therefore, in the bigger picture, our results align with the hypothesis of the involvement of a “salience network” in the apathy phenomenon [45,46,47].

Behavioural implementation is indeed a complex operator. Prefrontal regions, such as the lOFC and the vmPFC, are intertwined with the basal ganglia and the premotor cortex [42]. Interestingly, we did find that the PMC and the PSC are both associated with apathy in patients with bvFTD. This evidence highlights potential disturbances in the behavioural activation system, as proposed by Bonnelle et al. [48]. These authors investigated which brain regions were associated with more or less active behaviour in healthy individuals in response to a behavioural task. People with a higher level of baseline apathy had greater recruitment of the supplementary motor area (SMA) and the cingulate motor zones, suggesting a higher effort sensitivity. On the other hand, higher levels of baseline apathy were associated with decreased structural and functional connectivity between the ACC and the SMA. Given the above, it is reasonable to admit that a greater level of behavioural inactivation may be seen in patients due to impairments in the behavioural activation system, such as the ACC and OFC, as well as in motor areas. The rationale is that many “normal” situations may be inadvertently perceived as too risky or too effort-demanding [44,48]. This may be particularly true for the social dimension of apathy, a more recent category that still requires further investigation [4]. Kumfor et al. [11] studied apathy in patients with AD and bvFTD and found that affective apathy was more linked to the vmPFC, behavioural apathy with the basal ganglia, and cognitive apathy with the dmPFC. Their evidence shows that the right OFC was more associated with affective apathy, while the left OFC was related to behavioural apathy. In addition, emerging research highlights the cerebellum’s significant role in social cognition and empathy, which seems to be involved in processing social and emotional information. Unfortunately, we lack data on cerebellum structures but recommend encompassing this information in future investigations [49]. Additionally, future studies should consider the interface between certain subtypes of frontotemporal dementia, such as primary progressive aphasia and right temporal variant frontotemporal dementia (rtvFTD), as this may enhance the understanding of apathetic mechanisms [50].

Some limitations to our study must be acknowledged. First, a small sample of patients precluded further analysis and exploration of our data. Although common in the literature, due to the difficulty of recruiting bvFTD patients, we understand that a small sample can limit the power of statistical analysis. In addition, the choice of the SAS-C is debatable. We chose such a scale for several reasons, such as it being easy to answer and its use in different research settings in Brazil [51,52,53]. Conversely, evidence about SAS-C’s dimensions is not available in our context, and there are questions about its psychometric properties [54]. In addition, controlling for medication in the future may assist in better understanding the mechanisms of apathy. In our study, all patients enlisted with AD were undergoing treatment with acetylcholinesterase inhibitors, while some individuals with bvFTD were prescribed antipsychotics and atypical antidepressants, including trazodone. However, the precise proportion of patients using these medications cannot be determined retrospectively.

## 5. Conclusions

Our results confirm that apathy can be accurately assessed using a third-party questionnaire, reliably capturing both behavioural and neuroimaging correlates in AD and bvFTD. Furthermore, we provide clear evidence that apathy is linked to distinct brain regions in each disease, reinforcing the notion that AD and bvFTD exhibit specific apathetic profiles. In bvFTD, apathy also shows broader associations with cognitive functions compared to AD. Moving forward, we will expand our research to investigate subcortical structures and further refine the neural networks underlying apathy, as well as deepen our analysis of apathetic profiles in both current and future cohorts.

## Figures and Tables

**Table 1:** Demographical, clinical, neuropsychological, and behavioural results.

	Controls [*n* = 20]	AD [*n* = 19]	bvFTD [*n* = 20]	Statistical Group Comparison (η^2^ Effect Size)
Male/Female ratio	7:13	9:10	11:9	Not significant
Age (years), _mean (SD)_	63.9 (10.3)	70.4 (9.4)	64 (9.1)	Not significant
Education (years), _median (IQR)_	12.0 (4.0)	15.0 (5.0)	11.0 (4.0)	Not significant
Disease duration (years), _median (IQR)_	Not applicable	3.0 (1.0)	3.0 (2.0)	Not significant
MMSE, _median (IQR)_	29.0 (2.0)	24.0 (2.0)	26.0 (4.0)	Controls > all * (η^2^ = 0.58)
FAB, _median (IQR)_	16.0 (2.0)	14.0 (4.0)	12.6 (3.2)	Controls > all * (η^2^ = 0.27)
Fluency (FAS), _median (IQR)_	33.0 (10.0)	27.0 (19.0)	13.0 (6.0)	Controls = AD > bvFTD * (η^2^ = 0.30)
Fluency (Animals), _median (IQR)_	18.0 (4.1)	12.0 (8.0)	10.0 (6.0)	Controls > all * (η^2^ = 0.46)
FMT, Late recall, _median (IQR)_	9.0 (1.0)	4.0 (2.0)	7.0 (4.0)	Controls > all * (η^2^ = 0.52)
FMT, Recognition, _median (IQR)_	10.0 (0)	10.0 (1.0)	10.0 (0)	Not significant
Hayling Test				
- part A–time (s), _median (IQR)_	16.93 (7.79)	20.53 (8.42)	26.67 (20.45)	Controls < bvFTD * (η^2^ = 0.13)
- part B–time (s), _median (IQR)_	53.24 (51.78)	70.02 (32.71)	75.96 (83.94)	Not significant
- part B–score (PQt), _median (IQR)_	6.5 (5.0)	9.0 (3.0)	13.5 (5.0)	Controls < bvFTD * (η^2^ = 0.27)
- part B–scaled error (PQl), _median (IQR)_	9.0 (8.5)	15.0 (11.0)	33.5 (26.0)	Controls < bvFTD * (η^2^ = 0.26)
Faux-Pas Test (Total Score), _median (IQR)_	36.5 (5.0)	32.0 (5.0)	21.0 (13.0)	Controls = AD > bvFTD * (η^2^ = 0.63)
Ekman Total Score _median (IQR)_	28.0 (3.5)	26.0 (3.0)	21.5 (10.0)	Controls = AD > bvFTD * (η^2^ = 0.38)
Apathy score (SAS), _median (IQR)_	7.0 (5.50)	16.0 (10.0)	27.5 (12.0)	Controls > AD > bvFTD * (η^2^ = 0.64)

* Significant at *p* < 0.017. AD: Alzheimer’s disease; FAB: Frontal Assessment Battery; bvFTD: behavioural variant frontotemporal dementia; FMT: Figure Memory Test; MMSE: mini-mental state examination; SAS: Starkstein’s Apathy Scale.

## Data Availability

The raw data supporting the conclusions of this article will be made available by the authors on request.

## Supplementary Materials

The following are available online at https://www.mdpi.com/article/10.3390/brainsci15050447/s1, Figure S1: Receiver Operator Curve (ROC) analysis for SAS-C score in the clinical groups (AD x bvFTD); Table S1: Results for receiver operator characteristics curve analysis for apathy in the clinical groups (AD × bvFTD); Table S2: Spearman Correlation between Apathy score and brain regions (cortical thickness) in the Control group; Table S3: Spearman Correlation between Apathy score and brain regions (cortical thickness) in the bvFTD group; Table S4: Spearman Correlation between Apathy score and brain regions (cortical thickness) in the AD group; Table S5: Spearman Correlation between Apathy score and brain regions (cortical thickness) in the AD + Control group; Table S6: Spearman Correlation between Apathy score and brain regions in the bvFTD + Control group; Table S7: Multiple regression model outcomes for apathy and brain regions (cortical thickness)—AD + Controls; Table S8: Multiple regression model outcomes for apathy and brain regions (cortical thickness)—bvFTD + Controls.
