# Supplementary material for: Quantity and Quality Matter: Different Neuroanatomical Substrates of Apathy in Alzheimer’s Disease and Behavioural Variant Frontotemporal Dementia
Source: Brain Sci. 2025 Apr 25;15(5):447. doi: 10.3390/brainsci15050447 (PMC12110082; doi:10.3390/brainsci15050447)

SUPPLEMENTARY MATERIAL

Supplementary Table S1: Results for receiver operator characteristics curve analysis for apathy in the clinical groups (AD x bvFTD)

Area under the curve (AUC)	Standard Error[A]	Confidence Interval (95%)	p-value
**.805**	.074	.661 - .950	.001

A: under the non-parametric assumption.

Supplementary Table S2: Spearman Correlation between Apathy score and brain regions (cortical thickness) in the Control group

Cortical Region/Hemisphere	Left	Right
**Superior temporal sulcus**	-.321	-.166
**Caudal Anterior Cingulate**	-.360	.182
**Caudal Middle Frontal**	-.141	-.151
**Cuneus**	-.052	.061
**Entorhinal**	-.194	-.161
**Fusiform**	-.188	-.169
**Inferior Parietal**	-.081	-.134
**Inferior Temporal**	-.203	-.1
**Isthmus of cingulate gyrus**	-.292	-.353
**Lateral Occipital**	-.141	.032
**Lateral Orbitofrontal**	-.076	.017
**Lingual**	-.222	-.273
**Medial Orbitofrontal**	-.071	-.007
**Middle Temporal**	-.033	-.146
**Parahippocampal**	.085	-.022
**Paracentral**	-.169	-.086
**Pars opercularis**	-.023	-.008
**Pars orbitalis**	-.049	.075
**Pars triangularis**	-.066	-.062
**Pericalcarine**	-.102	.134
**Post central**	-.241	.121
**Posterior Cingulate**	-.193	-.021
**Pre central**	-.202	-.208
**Precuneus**	-.021	-.02
**Rostral Anterior Cingulate**	-.168	.135
**Rostral Middle Frontal**	-.092	-.125
**Superior Frontal**	.186	-.002
**Superior Parietal**	.111	-.131

**Superior Temporal**	-.176	-.277
**Supramarginal**	.133	-.017
**Frontal Pole**	.027	.159
**Temporal Pole**	.095	.1
**Transverse Temporal**	-.162	-.213
**Insula**	-.065	-.253

**** Significant at .05***

Supplementary Table S3: Spearman Correlation between Apathy score and brain regions (cortical thickness) in the bvFTD group

Cortical Region/Hemisphere	Left	Right
**Superior temporal sulcus**	.061	-.14
**Caudal Anterior Cingulate**	-.237	-.149
**Caudal Middle Frontal**	-.283	-.248
**Cuneus**	.025	-.056
**Entorhinal**	.064	.002
**Fusiform**	-.064	-.219
**Inferior Parietal**	.146	-.01
**Inferior Temporal**	-.067	-.01
**Isthmus of cingulate gyrus**	-.174	.214
**Lateral Occipital**	-.014	.288
**Lateral Orbitofrontal**	-.317	-.391
**Lingual**	-.053	-.017
**Medial Orbitofrontal**	-.202	-.454*
**Middle Temporal**	.089	-.097
**Parahippocampal**	.29	.061
**Paracentral**	-.088	-.113
**Pars opercularis**	-.414	-.362
**Pars orbitalis**	-.125	-.215
**Pars triangularis**	-.077	-.037
**Pericalcarine**	.146	.08
**Post central**	-.032	-.15
**Posterior Cingulate**	.076	.095
**Pre central**	-.235	-.07
**Precuneus**	-.22	-.019
**Rostral Anterior Cingulate**	-.353	-.02
**Rostral Middle Frontal**	-.159	-.396
**Superior Frontal**	-.2	-.091
**Superior Parietal**	-.342	-.283
**Superior Temporal**	.156	-.2

**Supramarginal**	-.181	-.33
**Frontal Pole**	-.042	-.109
**Temporal Pole**	-.03	.038
**Transverse Temporal**	-.442	-.395
**Insula**	-.149	-.247

** Significant at .05*

Supplementary Table S4: Spearman Correlation between Apathy score and brain regions (cortical thickness) in the AD group

Cortical Region/Hemisphere	Left	Right
**Superior temporal sulcus**	-.09	.1
**Caudal Anterior Cingulate**	.055	-.112
**Caudal Middle Frontal**	.079	.013
**Cuneus**	-.224	-.31
**Entorhinal**	-.435	-.387
**Fusiform**	-.139	-.18
**Inferior Parietal**	-.343	.099
**Inferior Temporal**	-.151	-.182
**Isthmus of cingulate gyrus**	-.223	-.302
**Lateral Occipital**	-.326	-.227
**Lateral Orbitofrontal**	.078	-.007
**Lingual**	.114	.238
**Medial Orbitofrontal**	-.245	-.37
**Middle Temporal**	.03	.121
**Parahippocampal**	-.474*	-.144
**Paracentral**	-.189	-.059
**Pars opercularis**	-.319	.008
**Pars orbitalis**	-.029	.052
**Pars triangularis**	-.214	-.1
**Pericalcarine**	-.326	-.168
**Post central**	-.247	.021
**Posterior Cingulate**	-.097	-.443
**Pre central**	-.086	-.005
**Precuneus**	-.325	-.191
**Rostral Anterior Cingulate**	-.059	.106
**Rostral Middle Frontal**	-.311	-.34
**Superior Frontal**	-.376	-.406
**Superior Parietal**	-.26	.128
**Superior Temporal**	-.529*	-.246

**Supramarginal**	-.252	-.001
**Frontal Pole**	-.286	-.178
**Temporal Pole**	-.473*	-.374
**Transverse Temporal**	-.346	-.075
**Insula**	-.114	-.539*

**** Significant at .05***

Supplementary Table S5: Spearman Correlation between Apathy score and brain regions (cortical thickness) in the AD+Control group

Cortical Region/Hemisphere	Left	Right
**Superior temporal sulcus**	-.513**	-.466**
**Caudal Anterior Cingulate**	-.246	-.183
**Caudal Middle Frontal**	-.24	-.426**
**Cuneus**	-.255	-.307
**Entorhinal**	-.602**	-.615**
**Fusiform**	-.524**	-.621**
**Inferior Parietal**	-.559**	-.453**
**Inferior Temporal**	-.489**	-.468**
**Isthmus of cingulate gyrus**	-.557**	-.567**
**Lateral Occipital**	-.458**	-.454**
**Lateral Orbitofrontal**	-.449**	-.448**
**Lingual**	-.284	-.276
**Medial Orbitofrontal**	-.457**	-.487**
**Middle Temporal**	-.549**	-.483**
**Parahippocampal**	-.547**	-.408**
**Paracentral**	-.351*	-.281
**Pars opercularis**	-.518**	-.323*
**Pars orbitalis**	-.401*	-.204
**Pars triangularis**	-.403*	-.394*
**Pericalcarine**	-.266	-.261
**Post central**	-.522**	-.341*
**Posterior Cingulate**	-.593**	-.574**
**Pre central**	-.443**	-.376*
**Precuneus**	-.524**	-.512**
**Rostral Anterior Cingulate**	-.338*	-.179
**Rostral Middle Frontal**	-.436**	-.509**
**Superior Frontal**	-.391*	-.457**
**Superior Parietal**	-.433**	-.392*
**Superior Temporal**	-.619**	-.527**
**Supramarginal**	-.435**	-.469**

**Frontal Pole**	-.415**	-.245
**Temporal Pole**	-.565**	-.489**
**Transverse Temporal**	-.380*	-.277
**Insula**	-.563**	-.631**

**** Significant at .05***
***** Significant at .01***

Supplementary Table S6: Spearman Correlation between Apathy score and brain regions in the bvFTD+Control group

Cortical Region/Hemisphere	Left	Right
**Superior temporal sulcus**	-.449**	-.457**
**Caudal Anterior Cingulate**	-.515**	-.278
**Caudal Middle Frontal**	-.492**	-.516**
**Cuneus**	-.042	-.137
**Entorhinal**	-.395*	-.584**
**Fusiform**	-.509**	-.514**
**Inferior Parietal**	-.327*	-.401*
**Inferior Temporal**	-.429**	-.460**
**Isthmus of cingulate gyrus**	-.368*	-.202
**Lateral Occipital**	-.272	-.195
**Lateral Orbitofrontal**	-.604**	-.640**
**Lingual**	-.222	-.199
**Medial Orbitofrontal**	-.583**	-.646**
**Middle Temporal**	-.552**	-.547**
**Parahippocampal**	-.28	-.488**
**Paracentral**	-.379*	-.422**
**Pars opercularis**	-.702**	-.583**
**Pars orbitalis**	-.394*	-.460**
**Pars triangularis**	-.665**	-.523**
**Pericalcarine**	-.002	-.007
**Post central**	-.254	-.3
**Posterior Cingulate**	-.524**	-.392*
**Pre central**	-.514**	-.502**
**Precuneus**	-.317*	-.274
**Rostral Anterior Cingulate**	-.676**	-.297
**Rostral Middle Frontal**	-.373*	-.546**
**Superior Frontal**	-.503**	-.602**
**Superior Parietal**	-.375*	-.448**
**Superior Temporal**	-.533**	-.598**
**Supramarginal**	-.425**	-.456**

**Frontal Pole**	-.341*	-.360*
**Temporal Pole**	-.453**	-.438**
**Transverse Temporal**	-.437**	-.510**
**Insula**	-.657**	-.679**

***** Significant at .05*
*** Significant at .01*

Supplementary Table S7: Multiple regression model outcomes for apathy and brain regions (cortical thickness) – AD+Controls:

Model		B	Standard Error	Beta	Significance
**1**	(Constant)	64.464	9.911		.000
	MMSE Total	-1.950**	.367	-.657	.000
**2**	(Constant)	64.398	10.107		.000
	MMSE Total	-1.964**	.434	-.662	.000
	FAB Total	.029	.487	.009	.952
**3**	(Constant)	62.807	10.254		.000
	MMSE Total	-1.947**	.435	-.656	.000
	FAB Total	.367	.602	.110	.546
	Verbal Fluency FAS	-.126	.131	-.160	.344
**4**	(Constant)	62.968	10.448		.000
	MMSE Total	-1.920**	.473	-.647	.000
	FAB Total	.415	.679	.125	.546
	Verbal Fluency FAS	-.121	.136	-.154	.379
	Faux-Pas Total Score	-.051	.320	-.032	.874
**5**	(Constant)	60.233	14.468		.000
	MMSE Total	-1.791*	.714	-.604	.017
	FAB Total	.422	.703	.127	.552
	Verbal Fluency FAS	-.122	.145	-.155	.409
	Faux-Pas Total Score	-.047	.333	-.029	.889
	TMF Late Recovery	-.194	.697	-.067	.782
	TMF Imediate Memory	.046	1.110	.010	.967

**6**	(Constant)	59.052	13.619		.000
	MMSE Total	-1.446*	.689	-.488	.044
	FAB Total	.268	.665	.081	.690
	Verbal Fluency FAS	-.137	.137	-.174	.324
	Faux-Pas Total Score	.114	.321	.071	.725
	TMF Late Recovery	.052	.664	.018	.938
	TMF Imediate Memory	-.203	1.050	-.045	.848
	Entorhinal (Left)	-9.876*	4.346	-.337	.030

a. Dependent variable: Apathy Total Score (SAS)
* p < .05 ǀ ** p < .001
R^2 = .432 for model 1 (significant at p < .001)
ΔR^2 = .000 for model 2 (not significant)
ΔR^2 = .0015 for model 3 (not significant)
ΔR^2 = .000 for model 4 (not significant)
ΔR^2 = .001 for model 5 (not significant)
ΔR^2 = .079 for model 6 (significant at p < .05)
AD: Alzheimer's disease
FAB: Frontal Assessment Battery
FMT: Figure Memory Test
MMSE: Mini Mental State Examination

Supplementary Table S8: Multiple regression model outcomes for apathy and brain regions (cortical thickness) – bvFTD+Controls

Model		B	Standard Error	Beta	Significance
**1**	(Constant)	92.577	12.789		.000
	MMSE Total	-2.802**	.468	-.707	.000
**2**	(Constant)	89.076	14.007		.000
	MMSE Total	-2.483**	.686	-.626	.001
	FAB Total	-.370	.578	-.111	.526
**3**	(Constant)	78.507	11.961		.000
	MMSE Total	-2.432**	.572	-.613	.000
	FAB Total	1.317	.637	.394	.046
	Verbal Fluency FAS	-.569**	.140	-.652	.000

**4**	(Constant)	68.436	11.229		.000
	MMSE Total	-1.788*	.555	-.451	.003
	FAB Total	1.772*	.591	.530	.005
	Verbal Fluency FAS	-.400	.138	-.459	.006
	Faux-Pas Total Score	-.625	.206	-.516	.005
**5**	(Constant)	76.072	13.154		.000
	MMSE Total	-2.134	.748	-.538	.008
	FAB Total	1.727	.597	.517	.007
	Verbal Fluency FAS	-.365	.147	-.419	.018
	Faux-Pas Total Score	-.670	.211	-.553	.003
	TMF Late Recovery	1.122	.849	.238	.196
	TMF Imediate Memory	-.760	1.161	-.116	.518
**6**	(Constant)	89.139	12.792		.000
	MMSE Total	-2.094	.677	-.528	.004
	FAB Total	1.243	.568	.372	.037
	Verbal Fluency FAS	-.346	.133	-.397	.014
	Faux-Pas Total Score	-.393	.215	-.324	.078
	TMF Late Recovery	.585	.792	.124	.466
	TMF Imediate Memory	-.050	1.081	-.008	.963
	Lateral Orbitofrontal (Right)	-3.904	1.396	-.281	.009

a. Dependent variable: Apathy Total Score (SAS)

* $p < .05$ | ** $p < .001$

$R^2 = .499$ for model 1 (significant at $p < .001$)

$\Delta R^2 = .006$ for model 2 (not significant)

$\Delta R^2 = .0161$ for model 3 (significant at $p < .001$)

$\Delta R^2 = .073$ for model 4 (significant at $p < .05$)

$\Delta R^2 = .014$ for model 5 (not significant)

$\Delta R^2 = .051$ for model 6 (significant at $p < .05$)

AD: Alzheimer's disease
FAB: Frontal Assessment Battery
FMT: Figure Memory Test
MMSE: Mini Mental State Examination

Supplementary Figure S1: Receiver Operator Curve (ROC) analysis for SAS-C score in the clinical groups (AD x bvFTD).

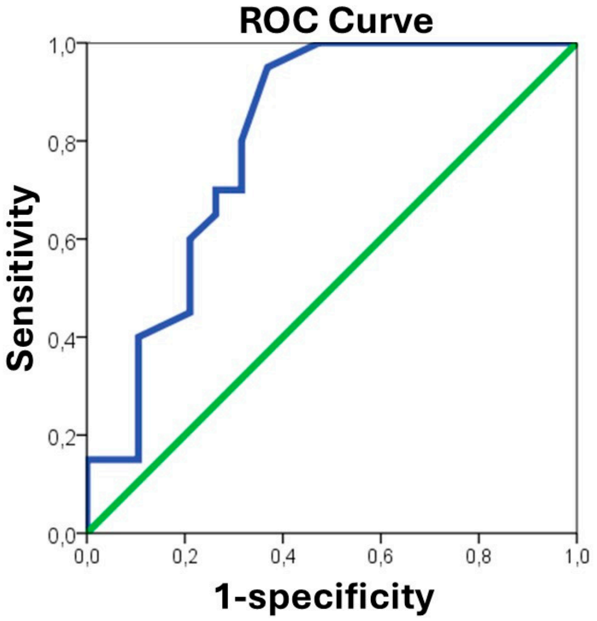

Supplement: Supplementary file 1 [file brainsci-15-00447-s001.zip › brainsci-3501971-supplementary.pdf]
